# Case Report: Abdominal Wall Reconstruction in a High-Risk Patient With Incisional Hernia and Complications From Oncological Treatment

**DOI:** 10.3389/jaws.2023.11767

**Published:** 2023-09-11

**Authors:** Thiago Souza Silva, Mario Rino Martins, Thales Lima Batista, Euclides Dias Martins, Marcelo Henrique Fernandes, Eduarda Araujo Hinrichsen

**Affiliations:** ^1^ Real Hospital Português, Recife, Brazil; ^2^ European Hernia Society (EHS), Gdansk, Poland; ^3^ Colégio Brasileiro de Cirurgiões, Rio de Janeiro, Brazil

**Keywords:** abdominal wall reconstruction, incisional hernia, hernia sac transposition, mesh-free technique, case report

## Abstract

**Introduction:** A high risk patient with evisceration underwent to abdominal wall reconstruction without mesh or drains. We present a case of a 62 years-old female patient with a significant medical history of Wilson’s disease-related hepatopathy Child-Pugh class B classification, sequelae of a stroke, and relevant surgical background including total hysterectomy, oophorectomy, and Hartmann’s procedure for ovarian neoplasm stage 3. The patient developed a large incisional hernia in the midline incision while undergoing Bevacizumab (Avastin) treatment for clinical oncology. During an attempt at skin closure due to erosion and necrosis, there was progressive deterioration leading to evisceration. We opted for abdominal wall reconstruction by transposing the hernia sac without using mesh and employing hemostatic powder (Arista) to mitigate the risk of bleeding in a high-risk patient due to recent bevacizumab use and hepatopathy. The patient had a favorable postoperative course without any other intervention in abdominal wall. Patient developed worsening hepatic function with the presence of ascites, constipation, and disorientation. On the 6th day postoperative, a tomography was performed, which showed colonic distension without obstructive factors and a slight amount of supra-aponeurotic fluid. The patient was discharged on the 10th day postoperative after improvement of the condition with clinical treatment. The patient has been progressing under outpatient follow-up for 5 months, with a resumption of chemotherapy cycles and no evidence of hernia recurrence.

**Conclusion:** Further studies and long-term follow-up are necessary to evaluate the efficacy and safety of hernia sac transposition as a mesh-free technique and the use of hemostatic powder without drains in high-risk patients. However, our case highlights the potential feasibility of these approaches in carefully selected cases.

## Introduction

Incisional hernia is a common complication following extensive abdominal surgeries [1]. The use of medications such as bevacizumab, an anti-angiogenic agent frequently employed in clinical oncology, may increase the risk of complications in abdominal wall repair [2]. We present a challenging case of abdominal wall reconstruction in a patient with multiple comorbidities and complications arising from oncological treatment.

## Case Presentation

**FIGURE 1:**
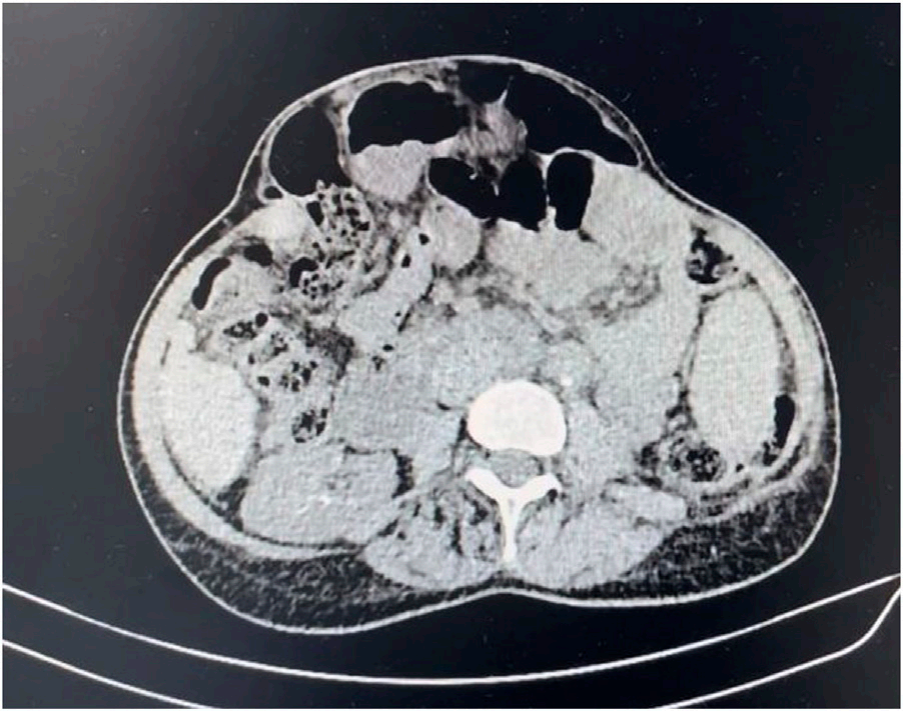
Preoperative tomography showing incisional hernia.

Female patient, 62 years old, with a surgical history of exploratory laparotomy, total hysterectomy, bilateral oophorectomy, and Hartmann’s sigmoidectomy for stage 3 ovarian neoplasia. After 2 years, she developed a 12 cm (length) × 10 cm (width), involving zones M2, M3, and M4 according to the EHS (European Hernia Society) classification, incisional hernia in the previous midline incision, observed on abdominal CT scan (Figure 1). The patient was under oncological follow-up and had been receiving a complex regimen, including the combination of carboplatin, paclitaxel, and bevacizumab. The patient underwent treatment cycles every 21 days. The patient was admitted to the emergency department after 6 days of chemotherapy infusion, presenting with ascites leakage through an area of ischemia and necrosis on the skin in the hernia sac region. This condition progressed to an expansion of the necrotic area and evisceration. The patient has other comorbidities, including secondary hepatopathy due to Wilson’s disease diagnosed 20 years ago, Child-Pugh class B at the time of admission, sequelae from a previous stroke, and osteoporosis.

**FIGURE 2:**
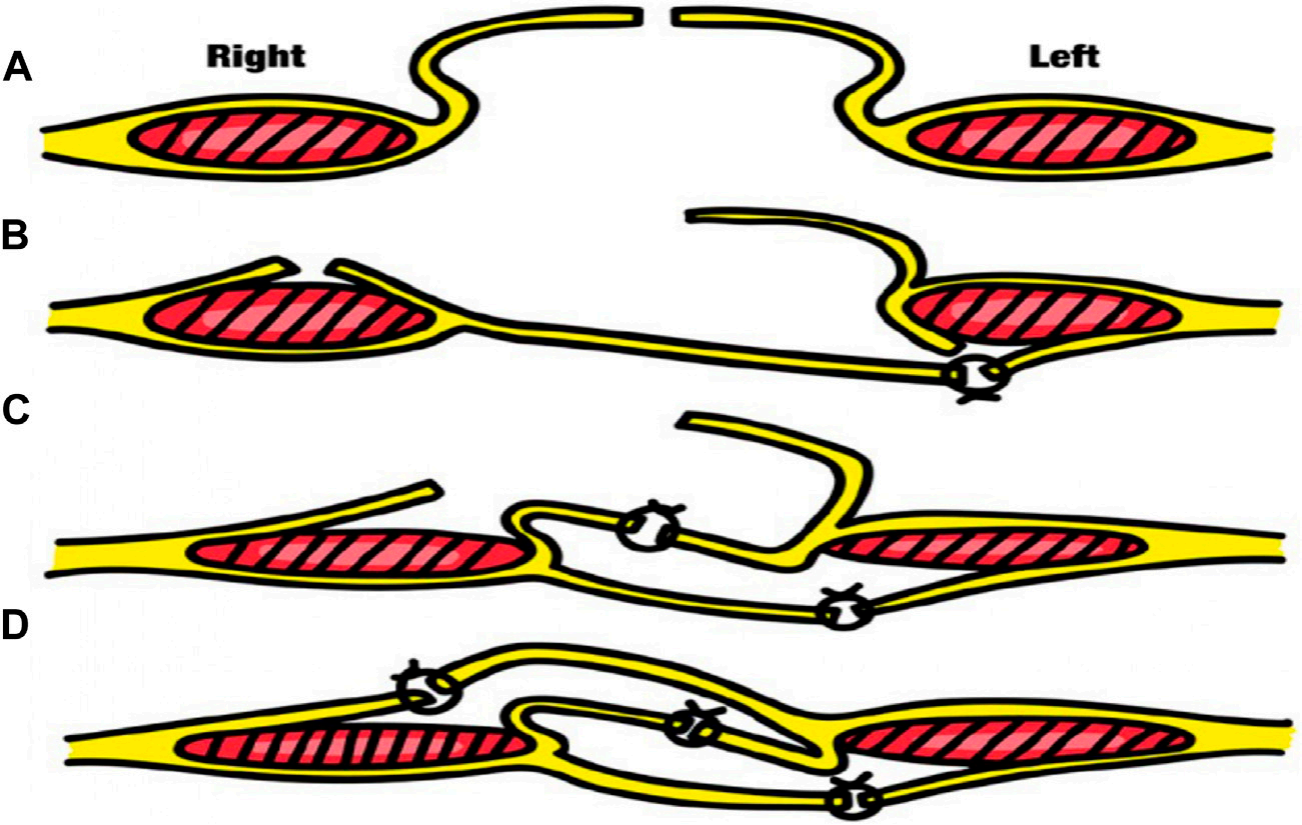
Transposition hernia Sac surgery technique – **(A)** - Opening of the hernia sac **(B)** - Anterior incision in the right rectal sheath and posterior incision in the left rectal sheath. **(C)** - Suturing the hernia sac flap to the right with the lateral portion of the left posterior rectal sheath and suturing the medial portion of the left rectal sheath to the medial suture of the right medial rectal sheath. **(D)** - Suturing the hernia sac flap to the left with the lateral portion of the right anterior rectal sheath.

**FIGURE 3:**
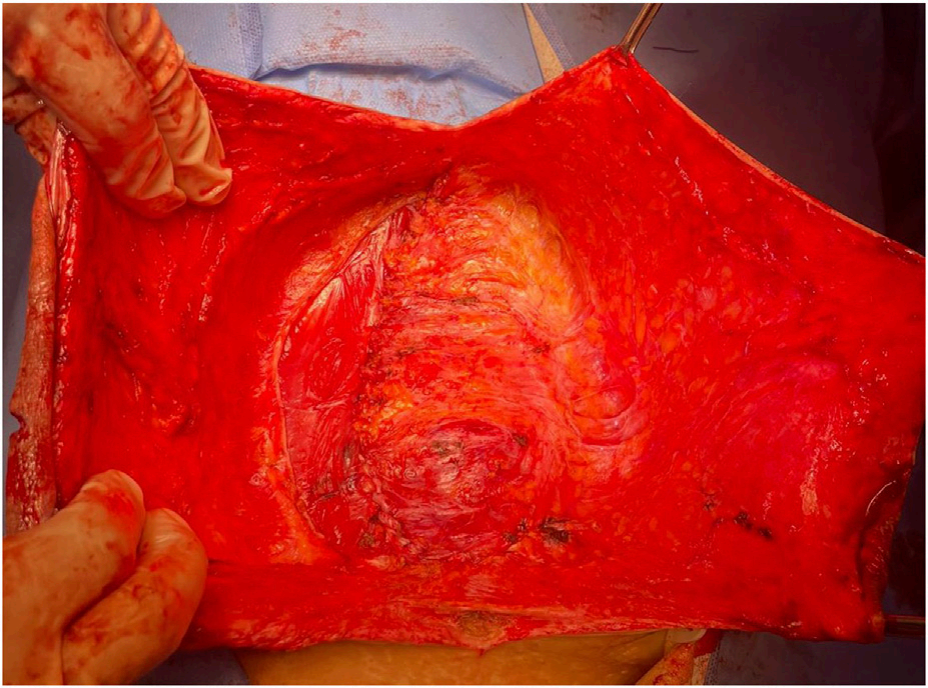
Final appearance of the surgery.

**FIGURE 4:**
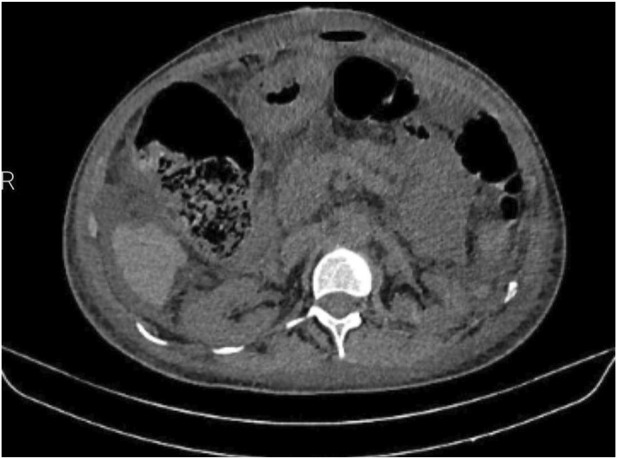
Abdominal tomography on the sixth postoperative day showing an intact abdominal wall with a small amount of supra-aponeurotic fluid.

The patient underwent the process of informed consent, being informed about the risks and possibilities of the proposed procedure. We opted for abdominal wall reconstruction by transposition of the hernia sac without mesh placement (Figures 2, 3) due to the high risk of abdominal wall complication as necrosis, bleeding and infection associated with recent bevacizumab use and hepatopathy. Additionally, Arista hemostatic powder was employed to assist in controlling bleeding and surgical site complications. The patient had postoperative recovery with no signs of dehiscence, surgical site infection or hematoma. A follow-up abdominal CT scan performed on the 6th postoperative day due to constipation and increased abdominal volume revealed mild inflammatory fluid and intestinal distension without signs of bleeding or obstructive factors (Figure 4). Clinical management of constipation and measures to address hepatic decompensation were instituted with a positive response. The patient was discharged without signs of dehiscence or hernia recurrence and has been under outpatient follow-up.

## Discussion

The management of complex incisional hernias in high-risk patients poses a challenge, particularly when considering the use of mesh in the presence of increased risks of bleeding, infection, and impaired wound healing [3]. In our case, we chose to perform abdominal wall reconstruction using mesh-free technique by transposing the hernia sac [4]. The technique involves performing three suture planes, where synthetic absorbable threads can be used, preferably long-lasting monofilaments with a caliber of 0 or even 2-0, in a simple continuous suture pattern. In the first plane, the free edge of the right half of the hernia sac (SH) is sutured to the left lateral posterior leaflet. Here, the peritoneal cavity is completely closed. A second plane is sutured between the middle leaflets, joining the right anterior and left posterior sheath, approximating the rectus abdominis muscles. Lastly, the edge of the left SH is sutured to the anterior leaflet. This bilateral muscle-aponeurotic advancement is only possible without excessive tension thanks to the two relaxing incisions made [5, 6]. This decision was based on the patient’s clinical conditions, which include the urgency of the surgery, hepatopathy, and recent use of a chemotherapy regimen involving bevacizumab. In this context, the risks of complications such as bleeding, infection, and necrosis are significant [7]. The transposition of the hernia sac technique offers several advantages in this scenario. By utilizing the intact sac as a barrier, the intra-abdominal contents can be effectively separated from the surgical wound, reducing the risk of contamination and subsequent infection. Moreover, this technique provides additional tissue coverage, which promotes wound healing and reduces the chances of dehiscence. This technique allows for an effective synthesis of the abdominal wall, with a shorter surgical time and reduced dissection area [5]. Some studies in high-risk patients reveals 7%–18% long-term recurrence rate with these surgical strategie. [6] In our experience for this patient profile, it emerges as a mesh-free option for abdominal reconstruction.

The decision to forgo the use of mesh was also influenced by the risk of infection associated with the recent use of bevacizumab, as this anti-angiogenic agent has been associated with impaired wound healing. By avoiding the placement of foreign material, we aimed to minimize the risk of mesh-related infection and further compromise the wound healing process.

In our case, we utilized hemostatic powder to manage intraoperative bleeding, thereby avoiding the use of drains. The application of hemostatic powder can provide effective control of bleeding and seroma, especially in patients at high risk for postoperative bleeding complications. This approach mitigates the need for drains, which have been associated with an increased risk of infection and discomfort [8].

Although the decision to omit mesh and drains in our patient’s abdominal wall reconstruction proved successful in terms of wound healing and absence of complications, it is important to acknowledge that each case should be evaluated individually. Factors such as the size and complexity of the hernia, the patient’s overall health status, and the surgeon’s expertise should be considered when taking into account mesh-free techniques and the omission of drains [3, 9].

Further studies and long-term follow-up are necessary to evaluate the efficacy and safety of hernia sac transposition without mesh and the use of hemostatic powder without drains in high-risk patients. However, our case highlights the potential feasibility of these approaches in carefully selected cases, particularly in patients with contraindications or heightened risks associated with mesh placement or drain usage.

In conclusion, our experience suggests that abdominal wall reconstruction with hernia sac transposition and the omission of mesh, along with the use of hemostatic powder without drains, can be a viable option in high-risk patients. Individualized decision-making, careful patient selection, and close postoperative monitoring are essential to achieve optimal outcomes in these challenging cases. Further research is warranted to establish standardized guidelines for such approaches in the management of complex incisional hernias.

## Data Availability

The original contributions presented in the study are included in the article/supplementary material, further inquiries can be directed to the corresponding author.
